# A Case of Oropharyngeal Bullous Pemphigoid Presenting with Haemoptysis

**DOI:** 10.1155/2015/631098

**Published:** 2015-01-06

**Authors:** C. M. Lee, H. K. Leadbetter, J. M. Fishman

**Affiliations:** ^1^Department of Dermatology, Royal Berkshire Hospital, London Road, Reading, Berkshire RG1 5AN, UK; ^2^Department of Ear, Nose and Throat Surgery, Royal Berkshire Hospital, Reading RG1 5AN, UK; ^3^University College London Ear Institute, 332 Grays Inn Road, London WC1X 8EE, UK

## Abstract

*Objective.* Bullous pemphigoid is well known for its cutaneous features; however in rare cases it may present with mucosal involvement. We report a case of bullous pemphigoid presenting with haemoptysis, initially presenting to the Ear, Nose and Throat Department for investigation. *Methods.* An 87-year-old lady was admitted with haemoptysis. She also complained of a spreading, pruritic, bullous rash, which first began three weeks previously. Initial investigations, which included nasendoscopy, revealed a normal nasal mucosa and a normal postnasal space. A large deroofed blister was observed on the soft palate. The presenting symptoms and signs raised the suspicion of an immunobullous disease including bullous pemphigoid. *Conclusion.* Bullous pemphigoid (BP) is a subepidermal immunobullous disease that typically manifests in elderly patient populations. Although rare, BP can present in a mucocutaneous fashion akin to its more aggressive variant, mucous membrane pemphigoid (MMP). Differentiation of the two is based on clinical grounds, with the prevailing feature for the latter being the predominance of mucosal involvement, which may be extensive. The mainstay of treatment for bullous pemphigoid is steroid therapy, which may be administered both topically and systemically. A deeper understanding into the pathophysiology of the various immunobullous diseases may assist in our understanding of how the various disease entities manifest themselves.

## 1. Introduction

Bullous pemphigoid is a subepidermal immunobullous disease most prevalent in elderly patients. Although rarely associated with mucosal lesions, bullous pemphigoid can present mucocutaneously. Mucocutaneous bullae may also raise the suspicion of mucous membrane (cicatricial) pemphigoid, a rare variant of bullous pemphigoid, as well as pemphigus vulgaris. There is frequently clinical overlap between these various conditions. Herein, we report a case of an 87-year-old lady, originally referred to us with haemoptysis, presenting with the concurrent development of tense skin blisters. The case was brought to the attention of the dermatologists in a prolonged hospital stay. This case demonstrates the value of histopathology in diagnosing immunobullous diseases. An understanding of the pathophysiology of the disease is essential for timely diagnosis.

## 2. Case Report

An 87-year-old lady was admitted to the ENT ward of a district general hospital with brisk, bright red haemoptysis. Subjectively, the patient felt the blood originated in her mouth and denied any coughing or vomiting, although she did complain of a sore throat.

She had been suffering intermittent haemoptysis for the past four months and had been investigated by multiple specialists who had not identified the source of the bleeding. She had been seen in ENT clinic, where serial nasendoscopy was normal. An oesophagogastroscopy failed to identify a cause for the bleeding. A subsequent CT of the thorax and abdomen was performed which only showed emphysematous lung changes without identifying a clear source to explain the haemorrhage. She also complained of a spreading, pruritic, blistering rash which had begun three weeks prior to presentation. She was on warfarin for atrial fibrillation, but on admission her INR was within therapeutic range (INR = 2.0).

**Figure 1:**
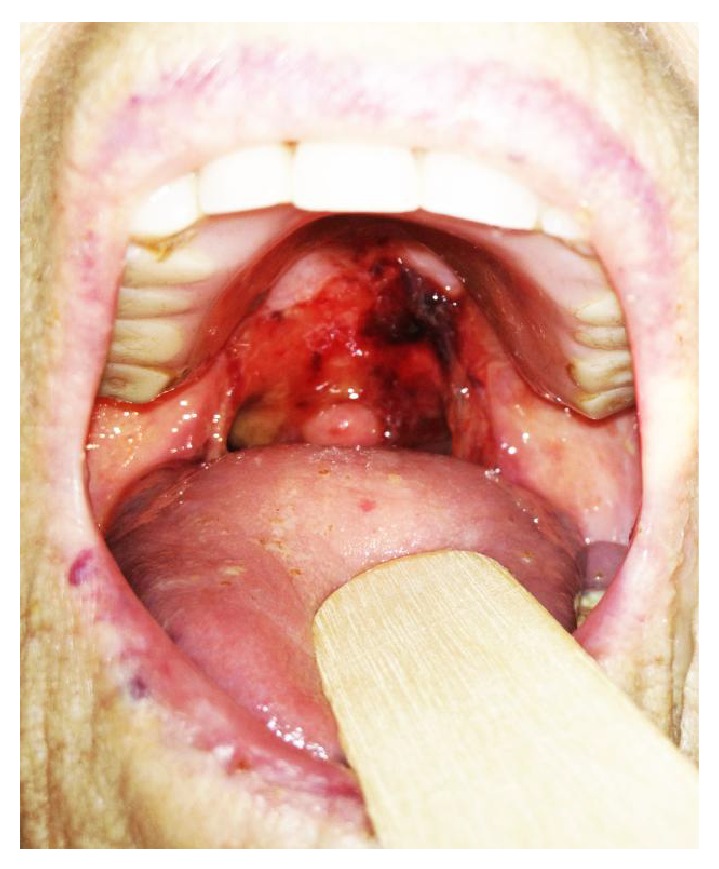
Haemorrhagic ulcer on soft palate resulting from a deroofed blister; in BP oral involvement is usually minor with blisters which break easily. Photograph was obtained with patient's written consent for publication.

On examination, the patient was seen spitting out bright red blood and a large deroofed blister was observed on the soft palate (Figure 1). Nasendoscopy revealed normal nasal mucosa and a normal postnasal space. Examination of her abdomen and respiratory systems was normal and there was no calf swelling or tenderness. She was noted to have multiple, annular ulcerated lesions on her back which the patient reported began as pruritic blisters which then ruptured. There were also some intact, tense blisters, overlying erythematous skin predominantly on the groins, but also on the arms, the inner aspect of left thigh, and right shin.

Blood tests demonstrated the presence of a mild microcytic anaemia and the persistence of a raised urea in keeping with swallowing of blood but were otherwise unremarkable.

The patient was subsequently referred to and reviewed by the Dermatology Team on the day of her admission. They felt that given her age and the presence of intact blisters that developed from pruritic lesions, this was clinically in keeping with bullous pemphigoid with mucous membrane involvement. The deroofing of blisters in the oropharynx could account for the intermittent haemoptysis and subsequent raised urea secondary to continuous ingestion of blood. Punch biopsies of perilesional skin were sent for histopathology, which confirmed the diagnosis of bullous pemphigoid, and the patient was commenced on both topical and systemic steroid therapies.

### 2.1. Investigations

**Figure 2:**
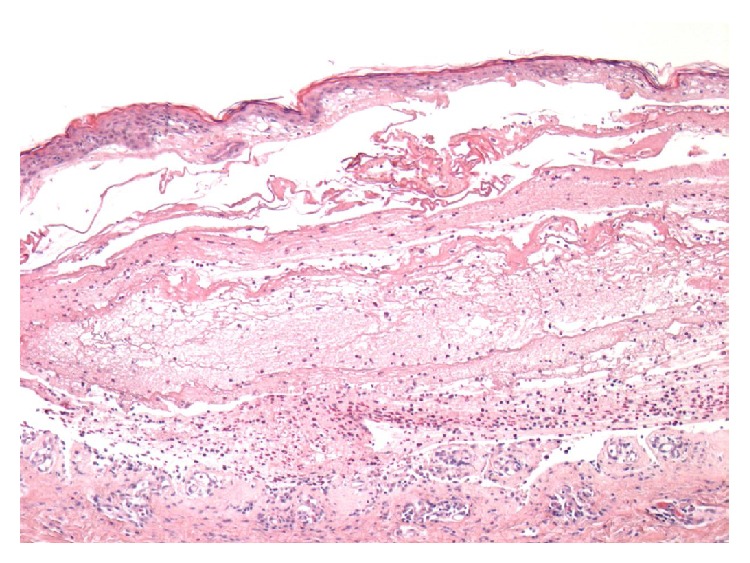
Histology demonstrating subepidermal blisters. The interface between epidermis and dermis layers is infiltrated by densely populated inflammatory cells around the basement membrane zone, including eosinophils (haematoxylin and eosin stain (magnification ×50)). Figure kindly provided by the Histopathology Department, Royal Berkshire Hospital, Reading, UK.

Histology demonstrated a subepidermal bulla containing fibrin and eosinophils, consistent with bullous pemphigoid (Figure 2). This diagnosis was supported by direct immunofluorescence, which showed linear homogenous deposition of IgG, C3, and IgA along the basement membrane zone (BMZ). IgM staining was negative. This pattern of linear staining is entirely in keeping with bullous pemphigoid.

The patient was commenced on systemic steroids, while awaiting biopsy reports, with prednisolone 30 mg once daily. This was a somewhat conservative dose prescribed in light of her comorbidities including congestive cardiac failure and ischaemic heart disease, compounded by mild anaemia, which at this point in her admission had decompensated to some extent, rendering her breathless. Potent topical steroids were applied to affected areas of skin and a steroid mouthwash was given. A daily blister chart was started to monitor the progress of therapy. Warfarin was withheld until her condition had improved.

Following one week of treatment, the disease activity appeared halted in its progression. After 2 weeks the systemic steroids were gradually tapered: the dose of prednisolone was reduced by 5 mg each week until a dose of 10 mg once daily was reached and thereafter weaned by 1 mg per week. Topical steroids were also reduced in strength gradually and were eventually stopped when no new blisters were reported.

The patient was followed up in outpatients clinic for several months, following the cessation of formation of new blisters at discharge. She had continued to complain of a sore mouth and was ultimately treated with a prolonged course of nystatin for oral, and probably oesophageal, candidiasis that occurred secondary to the long course of steroid therapy.

## 3. Discussion

From the outset, it was clear that the patient had an immunobullous disease. However, even in the presence of tense blisters and given the seniority of the patient's age, it was still challenging, nonetheless, to clinically differentiate between the various subepidermal immunobullous diseases. Indeed, there is frequently clinical overlap between these various conditions. Even though the gold standard test is histology with direct immunofluorescence, it is still challenging to classify the various immunobullous diseases because of our current incomplete identification of all those anchoring molecules within the basement membrane zone (BMZ) that are potentially targeted by autoantibodies. In addition, the mechanisms that lead to the formation of bullae are not fully understood.

Bullous pemphigoid (BP) is a subepidermal immunobullous disease of elderly people which typically starts with pruritic, erythematous lesions and progresses into the formation of large tense blisters where intact epidermis forms the roof. The predominant subclass of antibodies that react with the target molecules within the BMZ complex is IgG4 [1]. IgG1 and IgG2 may also be present alongside IgG4. It is worth noting that IgA is present in approximately 20% of cases of bullous pemphigoid and some cases with IgA deposition do have associated oral lesions [2].

Two principal hemidesmosomal proteins strongly linked to bullous pemphigoid have been identified: BPAg1, also named BP230 after its molecular weight of 230 kDa [3], and BPAg2, otherwise known as BP180 with a molecular weight of 180 kDa. The antigenic portions of BPAg1 are located at the carboxyl terminal end which is thought to play a role in interacting with keratin intermediate filaments in the BMZ [4]. The antigenic portion of BPAg2 molecule anchors extracellularly into the basement membrane bilayer (lamina lucida and lamina densa) and is located in the carboxyl terminal end [5].

As in this patient's case, bullous pemphigoid commonly begins with a nonspecific pruritic rash; the prodrome of itch and urticated plaques can last for several weeks before blisters form. Blisters are usually tense and contain clear serous exudates, which can occasionally be blood-stained, as in this case. Mucosal lesions can also occur; when they do, they tend to take a less aggressive course in BP compared to mucous membrane pemphigoid and pemphigus vulgaris and are usually confined to the oral cavity [6]. The blisters often remain intact for some time and if they rupture, resulting in erosions, they tend to heal fairly rapidly.

Positive direct and indirect immunofluorescence (IMF) play a central role in clinching a clinical and histological diagnosis. Since deposition of autoantibodies occurs in the BMZ, when the epidermis is lifted when taking a biopsy from the blister the immune-reactant may be lost from the roof of the blister. It is therefore best practice to take a biopsy from a perilesional site (within 2 cm of the lesion, where the skin appears normal). In direct IMF the biopsy will show either IgG or C3 along the BMZ; on occasions, as in this case, IgA and IgM may also occur. A serum sample is collected for indirect IMF, seeking a circulating IgG autoantibody against BMZ. Over the course of the disease, the predominant subclass of autoantibody switches from the more inflammatory IgG1 to the more downregulatory IgG4 with clinical attenuation of disease activity [7].

Without treatment, BP spontaneously resolves over several months to years. Relapses may occur, making up an average disease duration of around 6 years. Patients with BP, who are mostly elderly with comorbidities, have a twofold higher risk of death compared to the general population [8]. This case is an example of a BP patient, whose disease has unusually presented with predominantly mucosal involvement, thereby creating a diagnostic challenge. Bullous pemphigoid has been linked with a higher than normal incidence of dementia and this case also illustrates the importance of examining the patient fully to reveal skin lesions which may have been overlooked by both patient and previous clinicians. Although mucous membrane (cicatricial) pemphigoid has been previously reported to occur in the upper aerodigestive tract [9–11], BP, which is a clinically distinct condition, to our knowledge, has not previously been reported to occur in the oropharynx but appears to be as extensive as oesophageal involvement [12].

Steroids, both topical and systemic, aimed at suppressing disease activity with the minimal dose are necessary. The clinical disease is driven by circulating autoantibodies, thereby targeting various epitopes within the BMZ complex, on which they are deposited, and a prolonged course of treatment is often required, to suppress the lasting effect of the inflammatory cascade driven by complements, in particular C3. The recommended initial dose of prednisolone is 0.3 mg/kg/day in localised disease, 0.6 mg/kg/day in moderate disease, and 0.75–1 mg/kg/day in severe disease [13]. Once the disease activity is controlled (measured by the absence of appearance of new lesions, along with a fall of serum inflammatory markers) doses can be slowly withdrawn. In exceptional cases where disease control requires a prolonged course of moderate to high dose oral steroids, immunosuppressants can be introduced, with evidence supporting the adjunctive use of azathioprine or mycophenolate mofetil [14].

Finally, bullous pemphigoid tends to carry a good prognosis which has a low mortality with prompt diagnosis and treatment. Poor prognostic factors are age, generalised disease, low albumin, and high doses of steroids [15]. The major goals for treatment are to promote the arrest of new blister formation, encourage healing of existing lesions, and improve quality of life.

## 4. Key Points

- Bullous pemphigoid (BP) typically affects the skin whereas mucous membrane (cicatricial) pemphigoid typically involves and is confined to mucous membranes.
- Although mucous membrane (cicatricial) pemphigoid has been previously reported to occur in the upper aerodigestive tract, BP, which is a clinically distinct condition, has not previously been reported to occur in the oropharynx to the best of our knowledge.
- This case report highlights a case where a patient presenting with haemoptysis has clinical and histological evidence of BP rather than mucous membrane (cicatricial) pemphigoid.
